# Key design elements of successful acute ischemic stroke treatment trials

**DOI:** 10.1186/s42466-022-00221-9

**Published:** 2023-01-05

**Authors:** L. Yperzeele, A. Shoamanesh, Y. V. Venugopalan, S. Chapman, M. V. Mazya, M. Charalambous, V. Caso, W. Hacke, P. M. Bath, I. Koltsov

**Affiliations:** 1 Antwerp NeuroVascular Center and Stroke Unit, Department of Neurology, University Hospital Antwerp, Edegem, Belgium; 2 Translational Neurosciences Research Group, Faculty of Medicine and Health Sciences, University of Antwerp, Edegem, Belgium; 3 Division of Neurology, McMaster University / Population Health Research Institute, Hamilton, Canada; 4 Department of Neurology, All India Institute of Medical Sciences, New Delhi, India; 5 Department of Neurology, University of Virginia, Charlottesville, USA; 6 Department of Neurology, Karolinska University Hospital, Stockholm, Sweden; 7 Department of Clinical Neuroscience, Karolinska Institutet, Stockholm, Sweden; 8 Department of Rehabilitation Sciences, Cyprus University of Technology, Limassol, Cyprus; 9 Laboratory of Cognitive and Neurological Sciences, Neurology Unit, Faculty of Science and Medicine, University of Fribourg, Fribourg, Switzerland; 10 Stroke Unit, Santa Maria Della Misericordia Hospital, University of Perugia, Perugia, Italy; 11 Department of Neurology, Ruprechts Karl University, Heidelberg, Germany; 12 Stroke Trials Unit, Mental Health & Clinical Neuroscience, University of Nottingham, Nottingham, UK; 13 Cerebrovascular Diseases Laboratory, Pirogov Russian National Research Medical University, Moscow, Russia; 14 Neurology, Neurosurgery, and Medical Genetics Department, Pirogov Russian National Research Medical University, Moscow, Russia; 15 Neuroimmunology Department, Federal Center of Brain Research and Neurotechnologies, Moscow, Russia

**Keywords:** Acute stroke care, Randomized controlled trials, Trial design, Stroke, Acute stroke therapy, Stroke research

## Abstract

**Purpose:**

We review key design elements of positive randomized controlled trials (RCTs) in acute ischemic stroke (AIS) treatment and summarize their main characteristics.

**Method:**

We searched Medline, Pubmed and Cochrane databases for positive RCTs in AIS treatment. Trials were included if (1) they had a randomized controlled design, with (at least partial) blinding for endpoints, (2) they tested against placebo (or on top of standard therapy in a superiority design) or against approved therapy; (3) the protocol was registered and/or published before trial termination and unblinding (if required at study commencement); (4) the primary endpoint was positive in the intention to treat analysis; and (5) the study findings led to approval of the investigational product and/or high ranked recommendations. A topical approach was used, therefore the findings were summarized as a narrative review.

**Findings:**

Seventeen positive RCTs met the inclusion criteria. The majority of trials included less than 1000 patients (n = 15), had highly selective inclusion criteria (n = 16), used the modified Rankin score as a primary endpoint (n = 15) and had a frequentist design (n = 16). Trials tended to be national (n = 12), investigator-initiated and performed with public funding (n = 11).

**Discussion:**

Smaller but selective trials are useful to identify efficacy in a particular subgroup of stroke patients. It may also be of advantage to limit the number of participating countries and centers to avoid heterogeneity in stroke management and bureaucratic burden.

**Conclusion:**

The key characteristics of positive RCTs in AIS treatment described here may assist in the design of further trials investigating a single intervention with a potentially high effect size.

## Introduction

RCTs are needed to prove efficacy of a treatment in any disease, including stroke. RCTs can be big or small and in general trials with larger sample sizes are considered to yield higher success rates. In this article we review and discuss key design elements of positive acute ischemic stroke (AIS) trials and summarize their main characteristics.

## Methods

We searched Medline, Pubmed and Cochrane databases using the following search criteria: acute stroke treatment, neuroprotection, thrombolysis, mechanical thrombectomy, hemicraniectomy, and randomized. All papers published in English between 1980 and 2021 were evaluated for inclusion. The search was performed independently by WH, IK and AS.

Trials were considered ‘positive’ if they met the following criteria: (1) a randomized controlled design, with (at least partial) blinding for endpoints, (2) tested against placebo (or on top of standard therapy in a superiority design) or against approved therapy (such as rtPA); (3) protocol was registered and/or published before trial termination and unblinding (if required at study commencement); (4) primary endpoint was positive in the intention to treat analysis; and (5) the study findings led to approval of the investigational product and/or high ranked recommendations in internationally accepted guidelines.

Meta-analyses and pooled analyses of positive RCTs were not included. The few positive trials with a noninferiority design will be reported separately. Early secondary prevention trials were not considered.

As we used a topical approach, focusing on a general discussion of the subject instead of starting from a specific hypothesis, the findings were summarized in a narrative review.

## A short history of acute stroke treatment trials

### Neuroprotection

Neuroprotection aims to reduce the harmful effects of ischemia at the neuronal, glial, and blood–brain barrier level, and so limiting or even preventing tissue damage [1, 2]. Hundreds of candidate drugs have been tested in stroke models in rats, mice, canines and primates, many of them yielding smaller infarcts in treated animals. However, the positive effects were not confirmed in subsequent multicenter phase 2 and 3 clinical trials [3]. Reasons for the translation failure include: the use of inappropriate animal models (young, male animals), experiments which do not mimic clinical practice (e.g., treatment prior to vessel occlusion), use of different dosages, unexpected adverse events, overestimation of effect sizes leading to underpowered trials and wide eligibility criteria with inclusion of non-informative patients [4].

Only one RCT in neuroprotection showed positive results (SAINT-1 testing free-radical-trapping agent NXY-059), but the results were not confirmed in the SAINT-2 trial [5, 6]. The ESCAPE-NA1 (nerinetide in patients with a small ischemic core undergoing endovascular therapy) and URICO-ICTUS (combined uric acid and rtPA versus rtPA trials were neutral for their primary outcome but found positive signals in secondary endpoints or subgroups; however, a positive signal from a subgroup or a secondary outcome has never been confirmed in a subsequent stroke RCT [7, 8].

The Stroke Therapy Academic Industry Roundtable (STAIR) consortium has recommended several methodological improvements in further studies, including using drugs with multiple mechanisms, combining new interventions with thrombolytics, and using advanced imaging for patient-selection [9, 10]. Nevertheless, all trials following these approaches failed to find benefit, indicating that more rigorous preclinical designs are required before conducting phase 2 or 3 RCTs. Steps to improve the quality of experimental studies have been proposed by the Stroke Preclinical Assessment Network (SPAN) and several new compounds including fasudil (rho-kinase inhibitor), fingolimod (sphingosine-1-phosphate receptor inhibitor), tocilizumab (IL-6 antagonist), uric acid and veliparib (PARP-1 inhibitor) [11]. These should be tested in rigorous multicenter preclinical randomized blinded studies (as in clinical trials) involving more than one species and using clinically relevant outcomes and appropriate time windows [12].

### Antiplatelet therapy

Antiplatelet therapy might modulate the thrombotic event causing AIS, by reducing re-thrombosis and peripheral embolization. The Chinese Acute Stroke Trial (CAST) was positive, reporting that aspirin reduced all-cause death within four weeks (aspirin 3.3% vs control 3.9%, p = 0.044) [13]. The small effect size required that more than 20,000 patients would be needed. These results were supported by the just-neutral International Stroke Trial (IST), another aspirin mega-trial [14].

### Pharmacological recanalization

From 1994, several randomized, placebo-controlled, double-blind studies were conducted to determine the safety and efficacy of recombinant tissue plasminogen activator (rtPA) and streptokinase [15–24]. The first positive study, NINDS, was published in 1995 and showed that intravenous rtPA given between 0 to 3 h from stroke onset led to a 30% relative (11–15% absolute) increase in the number of patients with minimal or no disability at 90 days, compared to placebo [24]. Other RCTs published during the 1990s failed to reach their primary endpoint or showed an increased risk of intracranial hemorrhage. Methodological differences between the trials included the use of different thrombolytic agents and doses, variable treatment windows (e.g., up to 6 h) and concomitant use of antithrombotics [15, 16, 20–22].

A pooled analysis of these trials indicated efficacy up to 4.5 h without an increased risk of hemorrhage [18]. This prompted the design of the ECASS-3 trial which evaluated efficacy and safety of rtPA between 3 to 4.5 h and found favorable outcomes in patients treated with alteplase compared with placebo [19].

To further extend the treatment window, advanced magnetic resonance imaging was used in DIAS-1/2 and DEDAS trials of another thrombolytic agent, desmoteplase; the trials were neutral due to the presence of very small infarct cores [25–27].﻿ In 2018, the efficacy and safety of alteplase in wake-up stroke patients was demonstrated in the WAKE-UP study [28]. This led to the early termination of the EXTEND and the ECASS-4 trials which had selected patients using CT or MRI perfusion 4.5–9 h after onset or on awakening from stroke [29, 30].

### Mechanical recanalization

In addition to intravenous pharmacological reperfusion, intra-arterial revascularization has been explored, an approach originating in the positive PROACT-2 trial [31]. In 2004, the first Mechanical Embolus Removal in Cerebral Ischemia (MERCI) device for mechanical thrombectomy became commercially available, achieving around 50% recanalization rates in single arm open reports [32]. In 2009, the Penumbra system became available, combining aspiration-debulking with mechanical retrieval, which resulted in higher recanalisation rates (82% TIMI 2–3), but only a modest effect on clinical outcome (mRS 0–2 28% at 90 days) in another single arm study [33]. With self-expanding retrievable stents (Solitaire, Trevo) better radiographic and clinical outcomes were reported as compared to MERCI Retrievers [34, 35].

Subsequently, three trials (IMS-3, MR-RESCUE, SYNTHESIS) compared endovascular therapy with standard medical therapy and failed to show a significant difference in functional outcome (mRS 0–2) between the treatment groups [36–38]. However, the trials used first-generation devices and had slow recruitment, delayed times to reperfusion, and in one study, failed to demonstrate large-vessel occlusion before enrollment [39].

The MR-CLEAN trial finally confirmed the utility of mechanical thrombectomy versus standard care [40]. Several ongoing endovascular RCTs then stopped, showing positive results in the interim analyses. These trials used modern devices, achieved higher reperfusion rates, shorter onset-to-treatment time and used rtPA co-treatment in 75% of all cases [41–45]. Recently, the RESILIENT trial confirmed the feasibility and benefit of thrombectomy in a middle-income country [46].

The DAWN and DEFUSE-3 trials then confirmed the efficacy of thrombectomy in longer time windows (DAWN < 24 h, DEFUSE-3 < 16 h) using penumbra-infarct core imaging [47, 48].

## Findings

Seventeen stroke studies fulfilled the inclusion criteria for this review (Table 1): one trial in the field of neuroprotection (SAINT-1, although not confirmed in SAINT-2), one study of antiplatelet therapy (CAST); one hemicraniectomy trial (DESTINY-2); four trials of alteplase (NINDS, ECASS-3, WAKE-UP, EXTEND); one on intra-arterial thrombolysis (PROACT-2); and nine of mechanical thrombectomy (MR-CLEAN, SWIFT-PRIME, ESCAPE, EXTEND-IA, REVASCAT, THRACE, DEFUSE 3, DAWN, RESILIENT) [5, 13, 19, 24, 28, 29, 31, 40–49].

**Table 1:** Positive trials summary

Study, country, publication year	n	Endpoints^a^	Effect measure^b^	Primary sponsor	Termination	Inclusion criteria	Intervention	Control
NINDS [24] USA, 1995	624	Global test^c,d^	1.7 (1.2–2.6)	Public	Per protocol	Selective (time)	IVT	Placebo
CAST [13] China, 1997	21,106	28-day death Death or dependence at discharge	p = 0.04^e^ p = 0.08^e^	Public	Per protocol	Broad	Aspirin	Placebo
PROACT-2 [31] USA, 1999	180	mRS 0–2	p = 0.04^f^	Commercial	Per protocol	Selective (time, LVO)	IA prourokinase + heparin	Heparin
SAINT-1 [5] Global, 2006	1722	mRS shift	1.20^g^ (1.01–1.42)	Commercial	Per protocol	Selective (time), Broad (clinical)	Neuroprotection (NXY-059)	Placebo
ECASS-3 [19] European, 2008	821	mRS 0–1	1.42 (1.02–1.98)	Commercial	Per protocol	Selective (time)	IVT	Placebo
DESTINY-2 [49] Germany, 2014	112	mRS 0–4 at 6 months	2.91 (1.06–7.49)	Public	Per protocol	Selective (rare subgroup, imaging)	Hemicraniectomy	Conservative treatment
MR-CLEAN [40] Netherlands, 2015	500	mRS shift	1.67 (1.21–2.30)	Public	Per protocol	Selective (time, LVO)	MT + Standard care (90.6% IVT)	Standard care (87.1% IVT)
SWIFT-PRIME [45] Global, 2015	196	mRS shift mRS 0–2 (secondary)	p < 0.001^h^ RR 1.70 (1.23–2.33)	Commercial	Premature	Selective (time, LVO)	MT + IVT	IVT alone
ESCAPE [43] Canada, 2015	316	mRS shift	3.1 (2.0–4.7)	Commercial	Premature	Selective (time, LVO)	MT + Standard care (72.7% IVT)	Standard care (78.7% IVT)
EXTEND-IA [42] Australia, 2015	70	Reperfusion^i^ Early improvement^j^	4.7 (2.5–9.0) 6.0 (2.0–18.0)	Public	Premature	Selective (time, LVO, imaging)	MT + IVT	IVT alone
REVASCAT [44] Spain, 2015	206	mRS shift	1.7 (1.05–2.8)	Public	Premature	Selective (time, LVO)	MT + Standard care (68.0% IVT)	Standard care (77.7% IVT)
THRACE [41] France, 2016	414	mRS 0–2	1.55 (1.05–2.30)	Public	Premature	Selective (time, LVO)	MT + IVT	IVT alone
DEFUSE-3 [47] USA, 2018	182	mRS shift	Unadjusted OR 2.77 (1.63–4.70)	Public	Premature	Selective (time, LVO, imaging)	MT + Standard care	Standard care
DAWN [48] USA, 2018	206	Utility-weighted mRS % of mRS 0–2	AD^k^ 2.0 (1.1–3.0) AD^k^ 33 (21–44)	Commercial	Premature	Selective (time, LVO, imaging)	MT + Standard care (5.0% IVT)	Standard care (13.0% IVT)
WAKE-UP [28] European, 2018	503	mRS 0–1	1.61 (1.09–2.36)	Public	Premature	Selective (imaging)	IVT	Placebo
EXTEND [29] Australia, 2019	225	mRS 0–1	1.44 (1.01–2.06)	Public	Premature	Selective (time, imaging)	IVT	Placebo
RESILIENT [46] Brazil, 2020	221	mRS shift	2.28 (1.41–3.69)	Public	Premature	Selective (time, LVO)	MT + Standard care (68.5% IVT)	Standard care (71.8% IVT)

*AD*: adjusted difference; *IA*: intra-arterial; *IVT*: intravenous thrombolysis (alteplase); *LVO*: large vessel occlusion; *mRS*: modified Rankin Scale; *MT*: mechanical thrombectomy; OR: odds ratio; *RR*: risk ratio

^a^Primary or co-primary endpoints (at 90 days)

^b^Values presented are adjusted odds ratios with 95% confidence intervals

^c^Part 1 was negative, part 2 and combined results were positive

^d^Global test statistic for four primary outcome measures

^e^Two-sided p-value

^f^15% absolute increase in favorable outcome with intra-arterial prourokinase

^g^Effect was not reproduced in SAINT-2 (n = 3306, OR 0.94, 95% CI 0.83–1.06)

^h^Cochran-Mantel–Haenszel test

^i^Defined as the percentage reduction in the perfusion-lesion volume between initial imaging and 24-h imaging

^j^Defined as a reduction of 8 points or more on the National Institutes of Health Stroke Scale or a score of 0 or 1 at 3 days

^k^Bayesian trial design. For every co-primary outcome, adjusted differences and 95% credible intervals are presented

### Study size

Only two trials (SAINT-1 and CAST) included over 1000 patients [5, 13] Four trials included 500–1000 patients (NINDS, ECASS-3, MR-CLEAN, WAKE-UP), two trials included 250–499 patients (THRACE, ESCAPE) and nine trials included fewer than 250 patients (DESTINY-2, SWIFT-PRIME, EXTEND-IA, EXTEND, REVASCAT, DEFUSE-3, DAWN, RESILIENT, PROACT-2) [19, 24, 28, 29, 31, 40–49].

The majority of studies were halted prematurely, mainly due to loss of equipoise or overwhelming efficacy at interim analysis [28, 29, 41–48].

### Inclusion criteria: selective versus broad patient selection

While trials with broad inclusion criteria tend to be large and have long time windows, other trials have been defined based on: moderate to severe stroke symptoms, short onset-to-treatment times, localization of vessel occlusion, and/or advanced imaging selection. Except for CAST, all positive trials were highly selective [13].

### Study endpoints

Only two trials (EXTEND-IA, CAST) did not include the mRS score in its primary endpoint [13, 42]. The mRS was analyzed as a dichotomy (ECASS-3, DESTINY-2, THRACE, WAKE-UP, EXTEND, PROACT-2) or using its full distribution (SAINT-1, MR-CLEAN, SWIFT-PRIME, ESCAPE, REVASCAT, DEFUSE-3, RESILIENT) [5, 19, 28, 29, 31, 40, 41, 43–47, 49]. DAWN used the utility weighted mRS [48]. NINDS used a global endpoint encompassing the mRS, Barthel index, Glasgow outcome scale and NIHSS [24]. EXTEND-IA used recanalization and early NIHSS improvement as co-primary endpoints [42].

### Effect size

All but one trial used a frequentist design; DAWN used a Bayesian design [48]. With the exception of CAST, PROACT-2, SWIFT-PRIME and DAWN, most trials reported the odds ratio as the primary effect measure [13, 31, 45, 48]. The odds ratio was reported unadjusted in DEFUSE-3 and adjusted for covariates in 12 other trials [5, 19, 24, 28, 29, 40–44, 46, 47, 49]. Reported odds ratios ranged from 1.2 for modified Rankin scale shift in SAINT-1 [5] to 6.0 for early improvement in EXTEND-IA [42].

### Study origin and funding

Only three of the trials (SWIFT-PRIME, ESCAPE, SAINT-1) involved study sites on different continents [5, 43, 45]. Two trials recruited in several European countries (ECASS-3, WAKE-UP) [19, 28]. All other studies were national studies [13, 24, 29, 31, 40–42, 44, 46–49].

Two thirds of the trials were investigator initiated and publicly sponsored [13, 19, 28, 29, 40–42, 44, 46–49].

## Discussion

To the best of our ability, over the past 25 years, we were only able to identify 17 positive acute stroke trials With 888 interventional acute stroke trials registered in this period, the success rate is extremely low [50]; indeed there are more negative (not neutral) trials and interventions [3]. Successful trials share similarities in their design, most were investigator-initiated, publicly-funded, recruited nationally or regionally, and selected patients based on strict time, stroke severity, or imaging criteria. Half of these positive RCTs were ‘small’, with less than 250 patients. The first ever successful acute stroke intervention trial (NINDS) was a selective and small trial [24]. Between 1995 and 2014 only 5 positive trials were published [5, 19, 24, 31]. After the publication of MR-CLEAN and other thrombectomy trials, the number of positive RCTs increased, with 11 published between 2015 and 2021 [28, 29, 40–48].

In the design of clinical trials, there are two main scientific approaches. One advocates a pragmatic design with large sample size, many trial centers, broad patient eligibility criteria with a wide range of stroke severity and partial site monitoring. The other approach advocates for rigid selection criteria, strict data monitoring and standardization of interventions. The rationale for this latter approach is to include only patients likely to benefit from the intervention under investigation. Each approach may be right or wrong for a given scenario and careful consideration should be given to the approach to be used, although the track record of positive trials in acute stroke strongly speaks for the selective approach.

Based on the key characteristics of positive RCTs, several recommendations can be made for the design of acute stroke treatment trials. The effect size should reflect the expected mode of action and whether the intervention is expected to address multiple pathophysiological mechanisms. For example, malignant middle cerebral artery syndrome has a very poor outcome and hemicraniectomy is expected to dramatically reduce the risk of coning and death. Trials of hemicraniectomy are expected to be highly selective and small. In contrast, vessel occlusion by thrombus underlies many ischemic strokes, but has a complex pathophysiology; antithrombotic drugs are only likely to have a mild effect and so trials of aspirin (CAST, IST) had to be very large [13, 14].

Researchers should consider other clinical trials that address a similar research question, as publication of one positive trial can lead to loss of equipoise and premature termination of other trials. This is especially the case when results cause a paradigm shift, as was seen for thrombectomy after the publication of the results of MR CLEAN [40].

Phase III trials should be based on pilot studies that demonstrate proof of principle. Although it is tempting to design a trial on the basis of a positive subgroup in a neutral study, this approach has been unsuccessful in stroke trials. Positive subgroups may be falsely positive due to multiple statistical testing. Examples where a positive subgroup in one trial led to a neutral follow-on study include assessments of clomethiazole and piracetam for AIS, surgery for ICH, and glyceryl trinitrate for lowering blood pressure [51–58]. In contrast, adequately powered studies based on subgroup results from analyses of pooled trial data are more likely to be successful [19].

The mRS was the most common single primary outcome measure. The choice for using an ordinal (shift analysis) or simple dichotomized approach depends on the treatment effect of the intervention. Shift analysis is preferred when treatments yield a small, uniform degree of benefit over all ranges of stroke severity (f.ex. neuroprotection). When a substantial benefit over all ranges of stroke severity is expected, but treatment cannot provide a cure, dichotomization at good outcome is the most efficient statistical technique [59].

Trials tended to be investigator-initiated and performed with public funding or unconditional industry support, with investigators maintaining control of trial processes and publications. It may also be of advantage to limit the number of participating countries and centers so as to avoid heterogeneity in stroke management and bureaucratic burden, being mindful not to compromise the generalizability of the trial results.

However, this is not a one-size-fits-all model and these recommendations refer to hyperacute interventions in acute stroke and cannot be applied to prevention studies with much smaller effect sizes for which global trials involving thousands of patients are needed.

## Conclusions

In acute ischemic stroke research, smaller but selective trials are useful to identify efficacy in subgroups of stroke patients. The key characteristics of previous positive RCTs described here may assist in the design of further acute stroke treatment trials investigating a single intervention with a potentially high effect size.

## Data Availability

Not applicable.

## Abbreviations

AD: Adjusted difference
AIS: Acute ischemic stroke
CAST: Chinese Aspirin Stroke Trial
IA: Intra-arterial
IST: International Stroke Trial
IVT: Intravenous thrombolysis (alteplase)
LVO: Large vessel occlusion
MAST-I: Multicentre Acute Stroke Trial—Italy
MERCI: Mechanical Embolus Removal in Cerebral Ischemia
mRS: Modified Rankin Scale
MT: Mechanical thrombectomy
OR: Odds ratio
RCTs: Randomized controlled trials
RR: Risk ratio
rtPA: Recombinant tissue plasminogen activator
SPAN: Stroke Preclinical Assessment Network

## References

[1] Chamorro Á, Lo EH, Renú A, van Leyen K, Lyden PD (2021). The future of neuroprotection in stroke. Journal of Neurology, Neurosurgery, and Psychiatry.

[2] Paul S, Candelario-Jalil E (2021). Emerging neuroprotective strategies for the treatment of ischemic stroke: An overview of clinical and preclinical studies. Experimental Neurology.

[3] Bath PM, Appleton JP, England T (2020). The hazard of negative (not neutral) trials on treatment of acute stroke: A review. JAMA Neurology.

[4] Ginsberg MD (2008). Neuroprotection for ischemic stroke: past, present and future. Neuropharmacology.

[5] Lees KR, Zivin JA, Ashwood T, Davalos A, Davis SM, Diener H-C, Grotta J, Lyden P, Shuaib A, Hårdemark H-G, Wasiewski WW, Stroke-Acute Ischemic NXY Treatment (SAINT I) Trial Investigators (2006). NXY-059 for acute ischemic stroke. The New England Journal of Medicine.

[6] Shuaib A, Lees KR, Lyden P, Grotta J, Davalos A, Davis SM, Diener H-C, Ashwood T, Wasiewski WW, Emeribe U, Trial Investigators SAINTII (2007). NXY-059 for the treatment of acute ischemic stroke. The New England Journal of Medicine.

[7] Chamorro A, Amaro S, Castellanos M, Segura T, Arenillas J, Martí-Fábregas J, Gállego J, Krupinski J, Gomis M, Cánovas D, Carné X, Deulofeu R, Román LS, Oleaga L, Torres F, Planas AM, Investigators URICO-ICTUS (2014). Safety and efficacy of uric acid in patients with acute stroke (URICO-ICTUS): A randomised, double-blind phase 2b/3 trial. The Lancet. Neurology.

[8] Hill MD, Goyal M, Menon BK, Nogueira RG, McTaggart RA, Demchuk AM, Poppe AY, Buck BH, Field TS, Dowlatshahi D, van Adel BA, Swartz RH, Shah RA, Sauvageau E, Zerna C, Ospel JM, Joshi M, Almekhlafi MA, Ryckborst KJ, Lowerison MW, ESCAPE-NA1 Investigators (2020). Efficacy and safety of nerinetide for the treatment of acute ischaemic stroke (ESCAPE-NA1): A multicentre, double-blind, randomised controlled trial. Lancet (London, England).

[9] Albers GW, Goldstein LB, Hess DC, Wechsler LR, Furie KL, Gorelick PB, Hurn P, Liebeskind DS, Nogueira RG, Saver JL, STAIR VII Consortium (2011). Stroke Treatment Academic Industry Roundtable (STAIR) recommendations for maximizing the use of intravenous thrombolytics and expanding treatment options with intra-arterial and neuroprotective therapies. Stroke.

[10] Fisher M, Feuerstein G, Howells DW, Hurn PD, Kent TA, Savitz SI, Lo EH, STAIR Group (2009). Update of the stroke therapy academic industry roundtable preclinical recommendations. Stroke.

[11] *Stroke Pre-Clinical Assessment Network (SPAN)*. Retrieved November 11, 2021, from https://spannetwork.org/

[12] Bath PMW, Macleod MR, Green AR (2009). Emulating multicentre clinical stroke trials: A new paradigm for studying novel interventions in experimental models of stroke. International Journal of Stroke: Official Journal of the International Stroke Society.

[13] CAST (Chinese Acute Stroke Trial) Collaborative Group (1997). CAST: Randomised placebo-controlled trial of early aspirin use in 20,000 patients with acute ischaemic stroke. Lancet (London, England).

[14] International Stroke Trial Collaborative Group (1997). The International Stroke Trial (IST): A randomised trial of aspirin, subcutaneous heparin, both, or neither among 19435 patients with acute ischaemic stroke. Lancet (London, England).

[15] Clark WM, Albers GW, Madden KP, Hamilton S (2000). The rtPA (alteplase) 0- to 6-hour acute stroke trial, part A (A0276g): Results of a double-blind, placebo-controlled, multicenter study. Stroke.

[16] Clark WM, Wissman S, Albers GW, Jhamandas JH, Madden KP, Hamilton S (1999). Recombinant tissue-type plasminogen activator (Alteplase) for ischemic stroke 3 to 5 hours after symptom onset. The ATLANTIS study: A randomized controlled trial. Alteplase thrombolysis for Acute Noninterventional Therapy in Ischemic Stroke. JAMA.

[17] Donnan GA, Davis SM, Chambers BR, Gates PC, Hankey GJ, McNeil JJ, Rosen D, Stewart-Wynne EG, Tuck RR (1996). Streptokinase for acute ischemic stroke with relationship to time of administration: Australian Streptokinase (ASK) Trial Study Group. JAMA.

[18] Hacke W, Donnan G, Fieschi C, Kaste M, von Kummer R, Broderick JP, Brott T, Frankel M, Grotta JC, Haley EC, Kwiatkowski T, Levine SR, Lewandowski C, Lu M, Lyden P, Marler JR, Patel S, Tilley BC, Albers G, Bluhmki E, NINDS rt-PA Study Group Investigators (2004). Association of outcome with early stroke treatment: Pooled analysis of ATLANTIS, ECASS, and NINDS rt-PA stroke trials. Lancet (London, England).

[19] Hacke W, Kaste M, Bluhmki E, Brozman M, Dávalos A, Guidetti D, Larrue V, Lees KR, Medeghri Z, Machnig T, Schneider D, von Kummer R, Wahlgren N, Toni D, ECASS Investigators (2008). Thrombolysis with alteplase 3 to 4.5 hours after acute ischemic stroke. The New England Journal of Medicine.

[20] Hacke W, Kaste M, Fieschi C, Toni D, Lesaffre E, von Kummer R, Boysen G, Bluhmki E, Höxter G, Mahagne MH (1995). Intravenous thrombolysis with recombinant tissue plasminogen activator for acute hemispheric stroke. The European Cooperative Acute Stroke Study (ECASS). JAMA.

[21] Hacke W, Kaste M, Fieschi C, von Kummer R, Davalos A, Meier D, Larrue V, Bluhmki E, Davis S, Donnan G, Schneider D, Diez-Tejedor E, Trouillas P (1998). Randomised double-blind placebo-controlled trial of thrombolytic therapy with intravenous alteplase in acute ischaemic stroke (ECASS II). Lancet (London, England).

[22] Hommel M, Cornu C, Boutitie F, Boissel JP, Multicenter Acute Stroke Trial—Europe Study Group (1996). Thrombolytic therapy with streptokinase in acute ischemic stroke. The New England Journal of Medicine.

[23] Multicentre Acute Stroke Trial—Italy (MAST-I) Group (1995). Randomised controlled trial of streptokinase, aspirin, and combination of both in treatment of acute ischaemic stroke. Lancet (London, England).

[24] National Institute of Neurological Disorders and Stroke rt-PA Stroke Study Group (1995). Tissue plasminogen activator for acute ischemic stroke. The New England Journal of Medicine.

[25] Hacke W, Albers G, Al-Rawi Y, Bogousslavsky J, Davalos A, Eliasziw M, Fischer M, Furlan A, Kaste M, Lees KR, Soehngen M, Warach S (2005). DIAS Study Group. The Desmoteplase in Acute Ischemic Stroke Trial (DIAS): a phase II MRI-based 9-hour window acute stroke thrombolysis trial with intravenous desmoteplase. Stroke.

[26] Hacke W, Furlan AJ, Al-Rawi Y, Davalos A, Fiebach JB, Gruber F, Kaste M, Lipka LJ, Pedraza S, Ringleb PA, Rowley HA, Schneider D, Schwamm LH, Leal JS, Söhngen M, Teal PA, Wilhelm-Ogunbiyi K, Wintermark M, Warach S (2009). Intravenous desmoteplase in patients with acute ischaemic stroke selected by MRI perfusion-diffusion weighted imaging or perfusion CT (DIAS-2): a prospective, randomised, double-blind, placebo-controlled study. Lancet Neurology.

[27] Furlan AJ, Eyding D, Albers GW, Al-Rawi Y, Lees KR, Rowley HA, Sachara C, Soehngen M, Warach S, Hacke W (2006). DEDAS Investigators. Dose Escalation of Desmoteplase for Acute Ischemic Stroke (DEDAS): evidence of safety and efficacy 3 to 9 hours after stroke onset. Stroke.

[28] Thomalla G, Simonsen CZ, Boutitie F, Andersen G, Berthezene Y, Cheng B, Cheripelli B, Cho T-H, Fazekas F, Fiehler J, Ford I, Galinovic I, Gellissen S, Golsari A, Gregori J, Günther M, Guibernau J, Häusler KG, Hennerici M, Kemmling A, Marstrand J (2018). MRI-guided thrombolysis for stroke with unknown time of onset. New England Journal of Medicine.

[29] Ma H, Campbell BCV, Parsons MW, Churilov L, Levi CR, Hsu C, Kleinig TJ, Wijeratne T, Curtze S, Dewey HM, Miteff F, Tsai C-H, Lee J-T, Phan TG, Mahant N, Sun M-C, Krause M, Sturm J, Grimley R, Chen C-H, Hu C-J (2019). Thrombolysis guided by perfusion imaging up to 9 hours after onset of stroke. New England Journal of Medicine.

[30] Ringleb P, Bendszus M, Bluhmki E, Donnan G, Eschenfelder C, Fatar M, Kessler C, Molina C, Leys D, Muddegowda G, Poli S, Schellinger P, Schwab S, Serena J, Toni D, Wahlgren N, Hacke W, ECASS-4 study group (2019). Extending the time window for intravenous thrombolysis in acute ischemic stroke using magnetic resonance imaging-based patient selection. International Journal of Stroke: Official Journal of the International Stroke Society.

[31] Furlan A, Higashida R, Wechsler L, Gent M, Rowley H, Kase C, Pessin M, Ahuja A, Callahan F, Clark WM, Silver F, Rivera F (1999). Intra-arterial prourokinase for acute ischemic stroke. The PROACT II study: A randomized controlled trial. Prolyse in Acute Cerebral Thromboembolism. JAMA.

[32] Gobin YP, Starkman S, Duckwiler GR, Grobelny T, Kidwell CS, Jahan R, Pile-Spellman J, Segal A, Vinuela F, Saver JL (2004). MERCI 1: a phase 1 study of mechanical embolus removal in cerebral ischemia. Stroke.

[33] Penumbra Pivotal Stroke Trial Investigators (2009). The penumbra pivotal stroke trial: Safety and effectiveness of a new generation of mechanical devices for clot removal in intracranial large vessel occlusive disease. Stroke.

[34] Nogueira RG, Lutsep HL, Gupta R, Jovin TG, Albers GW, Walker GA, Liebeskind DS, Smith WS, TREVO 2 Trialists (2012). Trevo versus Merci retrievers for thrombectomy revascularisation of large vessel occlusions in acute ischaemic stroke (TREVO 2): A randomised trial. Lancet (London, England).

[35] Saver JL, Jahan R, Levy EI, Jovin TG, Baxter B, Nogueira RG, Clark W, Budzik R, Zaidat OO, Trialists SWIFT (2012). Solitaire flow restoration device versus the Merci Retriever in patients with acute ischaemic stroke (SWIFT): A randomised, parallel-group, non-inferiority trial. Lancet (London, England).

[36] Broderick JP, Palesch YY, Demchuk AM, Yeatts SD, Khatri P, Hill MD, Jauch EC, Jovin TG, Yan B, Silver FL, von Kummer R, Molina CA, Demaerschalk BM, Budzik R, Clark WM, Zaidat OO, Malisch TW, Goyal M, Schonewille WJ, Mazighi M, Engelter ST, Interventional Management of Stroke (IMS) III Investigators (2013). Endovascular therapy after intravenous t-PA versus t-PA alone for stroke. The New England Journal of Medicine.

[37] Ciccone A, Valvassori L, Nichelatti M, Sgoifo A, Ponzio M, Sterzi R, Boccardi E (2013). Endovascular treatment for acute ischemic stroke. New England Journal of Medicine.

[38] Kidwell CS, Jahan R, Gornbein J, Alger JR, Nenov V, Ajani Z, Feng L, Meyer BC, Olson S, Schwamm LH, Yoo AJ, Marshall RS, Meyers PM, Yavagal DR, Wintermark M, Guzy J, Starkman S, Saver JL (2013). A trial of imaging selection and endovascular treatment for ischemic stroke. New England Journal of Medicine.

[39] Goyal M, Almekhlafi M, Menon B, Hill M, Fargen K, Parsons M, Bang OY, Siddiqui A, Andersson T, Mendes V, Davalos A, Turk A, Mocco J, Campbell B, Nogueira R, Gupta R, Murphy S, Jovin T, Khatri P, Miao Z, Demchuk A (2014). Challenges of acute endovascular stroke trials. Stroke.

[40] Berkhemer OA, Fransen PSS, Beumer D, van den Berg LA, Lingsma HF, Yoo AJ, Schonewille WJ, Vos JA, Nederkoorn PJ, Wermer MJH, van Walderveen MAA, Staals J, Hofmeijer J, van Oostayen JA, Lycklama à Nijeholt GJ, Boiten J, Brouwer PA, Emmer BJ, de Bruijn SF, van Dijk LC, Kappelle LJ (2015). A Randomized Trial of Intraarterial Treatment for Acute Ischemic Stroke. New England Journal of Medicine.

[41] Bracard S, Ducrocq X, Mas JL, Soudant M, Oppenheim C, Moulin T, Guillemin F, THRACE investigators (2016). Mechanical thrombectomy after intravenous alteplase versus alteplase alone after stroke (THRACE): A randomised controlled trial. The Lancet. Neurology.

[42] Campbell BCV, Mitchell PJ, Kleinig TJ, Dewey HM, Churilov L, Yassi N, Yan B, Dowling RJ, Parsons MW, Oxley TJ, Wu TY, Brooks M, Simpson MA, Miteff F, Levi CR, Krause M, Harrington TJ, Faulder KC, Steinfort BS, Priglinger M, Ang T (2015). Endovascular therapy for ischemic stroke with perfusion-imaging selection. New England Journal of Medicine.

[43] Goyal M, Demchuk AM, Menon BK, Eesa M, Rempel JL, Thornton J, Roy D, Jovin TG, Willinsky RA, Sapkota BL, Dowlatshahi D, Frei DF, Kamal NR, Montanera WJ, Poppe AY, Ryckborst KJ, Silver FL, Shuaib A, Tampieri D, Williams D, Bang OY (2015). Randomized assessment of rapid endovascular treatment of ischemic stroke. New England Journal of Medicine.

[44] Jovin TG, Chamorro A, Cobo E, de Miquel MA, Molina CA, Rovira A, San Román L, Serena J, Abilleira S, Ribó M, Millán M, Urra X, Cardona P, López-Cancio E, Tomasello A, Castaño C, Blasco J, Aja L, Dorado L, Quesada H, Rubiera M (2015). Thrombectomy within 8 hours after symptom onset in ischemic stroke. New England Journal of Medicine.

[45] Saver JL, Goyal M, Bonafe A, Diener H-C, Levy EI, Pereira VM, Albers GW, Cognard C, Cohen DJ, Hacke W, Jansen O, Jovin TG, Mattle HP, Nogueira RG, Siddiqui AH, Yavagal DR, Baxter BW, Devlin TG, Lopes DK, Reddy VK, Jahan R (2015). Stent-retriever thrombectomy after intravenous t-PA vs T-PA alone in stroke. New England Journal of Medicine.

[46] Martins SO, Mont’Alverne F, Rebello LC, Abud DG, Silva GS, Lima FO, Parente BSM, Nakiri GS, Faria MB, Frudit ME, de Carvalho JJF, Waihrich E, Fiorot JA, Cardoso FB, Hidalgo RCT, Zétola VF, Carvalho FM, de Souza AC, Dias FA, Nogueira RG (2020). Thrombectomy for stroke in the public health care system of Brazil. New England Journal of Medicine.

[47] Albers GW, Marks MP, Kemp S, Christensen S, Tsai JP, Ortega-Gutierrez S, McTaggart RA, Torbey MT, Kim-Tenser M, Leslie-Mazwi T, Sarraj A, Kasner SE, Ansari SA, Yeatts SD, Hamilton S, Mlynash M, Heit JJ, Zaharchuk G, Kim S, Carrozzella J, Palesch YY (2018). Thrombectomy for Stroke at 6 to 16 Hours with Selection by Perfusion Imaging. New England Journal of Medicine.

[48] Nogueira RG, Jadhav AP, Haussen DC, Bonafe A, Budzik RF, Bhuva P, Yavagal DR, Ribo M, Cognard C, Hanel RA, Sila CA, Hassan AE, Millan M, Levy EI, Mitchell P, Chen M, English JD, Shah QA, Silver FL, Pereira VM, Mehta BP (2018). Thrombectomy 6 to 24 Hours after Stroke with a Mismatch between Deficit and Infarct. New England Journal of Medicine.

[49] Jüttler E, Unterberg A, Woitzik J, Bösel J, Amiri H, Sakowitz OW, Gondan M, Schiller P, Limprecht R, Luntz S, Schneider H, Pinzer T, Hobohm C, Meixensberger J, Hacke W (2014). Hemicraniectomy in older patients with extensive middle-cerebral-artery stroke. New England Journal of Medicine.

[50] NIH U.S. National Library of Medicine. *ClinicalTrials.gov*. Retrieved January 6, 2022, from https://clinicaltrials.gov/

[51] De Deyn PP, Reuck JD, Deberdt W, Vlietinck R, Orgogozo JM (1997). Treatment of acute ischemic stroke with piracetam. Stroke.

[52] ENOS Trial Investigators (2015). Efficacy of nitric oxide, with or without continuing antihypertensive treatment, for management of high blood pressure in acute stroke (ENOS): A partial-factorial randomised controlled trial. Lancet (London, England).

[53] Lyden P, Shuaib A, Ng K, Levin K, Atkinson RP, Rajput A, Wechsler L, Ashwood T, Claesson L, Odergren T, Salazar-Grueso E, CLASS-I, H, T Investigators (2002). Clomethiazole acute stroke study in ischemic stroke (CLASS-I): Final results. Stroke.

[54] Mendelow AD, Gregson BA, Fernandes HM, Murray GD, Teasdale GM, Hope DT, Karimi A, Shaw MDM, Barer DH, STICH investigators (2005). Early surgery versus initial conservative treatment in patients with spontaneous supratentorial intracerebral haematomas in the International Surgical Trial in Intracerebral Haemorrhage (STICH): A randomised trial. Lancet (London, England).

[55] Mendelow AD, Gregson BA, Rowan EN, Murray GD, Gholkar A, Mitchell PM, Investigators STICHII (2013). Early surgery versus initial conservative treatment in patients with spontaneous supratentorial lobar intracerebral haematomas (STICH II): A randomised trial. Lancet (London, England).

[56] Ricci S, Celani MG, Cantisani TA, Righetti E (2012). Piracetam for acute ischaemic stroke. The Cochrane Database of Systematic Reviews.

[57] RIGHT-2 Investigators (2019). Prehospital transdermal glyceryl trinitrate in patients with ultra-acute presumed stroke (RIGHT-2): An ambulance-based, randomised, sham-controlled, blinded, phase 3 trial. Lancet (London, England).

[58] Wahlgren NG, Ranasinha KW, Rosolacci T, Franke CL, van Erven PM, Ashwood T, Claesson L (1999). Clomethiazole acute stroke study (CLASS): Results of a randomized, controlled trial of clomethiazole versus placebo in 1360 acute stroke patients. Stroke.

[59] Saver JL, Gornbein J (2009). Treatment effects for which shift or binary analyses are advantageous in acute stroke trials. Neurology.

